# Surface Quality of 3D-Printed Models as a Function of Various Printing Parameters

**DOI:** 10.3390/ma12121970

**Published:** 2019-06-19

**Authors:** Christin Arnold, Delf Monsees, Jeremias Hey, Ramona Schweyen

**Affiliations:** Department of Prosthetic Dentistry, University School of Dental Medicine, Martin-Luther-University Halle-Wittenberg, Magdeburger Str. 16, 06112 Halle, Germany; christin.arnold@uk-halle.de (C.A.); delf.monsees@organical-cadcam.com (D.M.); jeremias.hey@uk-halle.de (J.H.)

**Keywords:** 3D-printing, surface roughness, support structure, layer height, dental model

## Abstract

Although 3D-printing is common in dentistry, the technique does not produce the required quality for all target applications. Resin type, printing resolution, positioning, alignment, target structure, and the type and number of support structures may influence the surface roughness of printed objects, and this study investigates the effects of these variables. A stereolithographic data record was generated from a master model. Twelve printing processes were executed with a stereolithography Desktop 3D Printer, including models aligned across and parallel to the printer front as well as solid and hollow models. Three layer thicknesses were used, and in half of all processes, the models were inclined at 15°. For comparison, eight gypsum models and milled polyurethane models were manufactured. The mean roughness index of each model was determined with a perthometer. Surface roughness values were approximately 0.65 µm (master), 0.87–4.44 µm (printed), 2.32–2.57 µm (milled), 1.72–1.86 µm (cast plaster/alginate casting), and 0.98–1.03 µm (cast plaster/polyether casting). The layer height and type and number of support structures influenced the surface roughness of printed models (*p* ≤ 0.05), but positioning, structure, and alignment did not.

## 1. Introduction

Computer-assisted additive manufacturing has become increasingly useful in medical and dental applications [1,2,3,4,5]. Numerous 3D printers based on different technologies are commercially available [6]: PolyJet, ColorJet, and fused deposition modeling are comparatively expensive technologies, whereas printers based on digital light projection and stereolithography (SLA) printers are relatively inexpensive. SLA printers in particular often serve as a user’s gateway to 3D printing. 

In dentistry, SLA printers are currently used to fabricate situational models, precision or saw-cut models, and models for implant works, as well as rails, implant drilling templates, functional trays, and even temporary dentures or complete denture prostheses [4]. Dental manufacturers promote SLA printers as a quick and easy means of fabricating many situational and saw-cut models on one build platform, thereby offering a significant reduction in the burden on dental laboratory personnel. 

However, for precision or saw-cut models, each patient’s oral cavity must be mapped with high accuracy and surface quality to produce high-precision fixed partial dentures. Previous studies investigating the required precision level in surface qualities’ reproduction recommended an accuracy in reproduction of 0.5 to 1 µm [7]. Conventionally fabricated precision models, transferred from polyether impressions, meet these requirements [7]. In contrast, other dental applications, such as situational models conventionally fabricated from alginate gypsum and providing surface roughnesses of 2 to 4 µm, do not require such high precision [7,8,9]. Printed 3D objects are known to not always meet these quality standards [10].

SLA printers contain a pool filled with synthetic resin. The bottom of the pool is transparent, and the inside surface is not highly adhesive. During the printing process, a build platform is gradually drawn out of the pool, followed by successive layers, each cured in turn by a laser beam directed through the bottom of the printer. The laser is deflected in a Cartesian coordinate system using mirror galvanometers [11,12]. The combined vertical movement of the build platform and horizontal or tilting movements of the pool release each newly created layer from the pool base. The layering process is repeated until the object is completely formed. However, freshly printed objects need post-treatment to attain their maximum strength [13].

The quality of an object produced by a SLA printer is influenced by many different parameters. Resolution plays a crucial role and is dependent on two factors: the layer height Z, defined as the distance between the build platform or the previously completed layer and the pool base; and the minimum structural dimensions that can be achieved in the XY coordinate plane with the available laser beam diameter. The composition of the synthetic resin also influences factors affecting quality, such as the grain size, viscosity, and reactivity. Other relevant parameters include the exposure time of the individual layers (especially that of the transition layer between the base layer and first layer), haul-off speed of the build platform from the pool base, and temperature of the pool bath. Manufacturers provide recommendations for optimizing these parameters. 

However, the literature indicates that additional factors, such as the angulation of the model, its position on the building platform, and whether the target structure is a solid or hollow body, also strongly influence the model accuracy and production time, thereby influencing cost [14,15]. Staircase effects on the surface [15,16,17,18], overheating, and distortions in overhang areas have been attributed to these factors [18].

This study investigates the effects of layer height, model positioning and alignment, type and number of support structures, and body type of the target structure on the surface quality of SLA-printed dental models. In order to set the surface quality of these models in relation to established standards, milled CAD (Computer Aided Design) models and conventionally manufactured models were included as reference groups.

As a null hypothesis, the surface quality of the printed models is assumed to be independent of the printing variables mentioned above and that the quality level of established model fabrication technologies, such as milled CAD or conventional manufacturing, can be achieved. 

## 2. Materials and Methods

**Figure 1:**
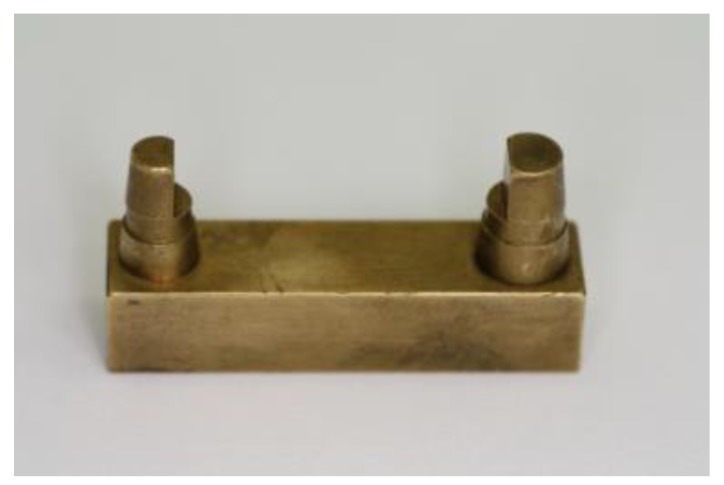
Master model.

The basis of the tests was a master model made of brass (Figure 1). The master model represents a stump situation for a single-span, four-unit bridge (pillars: canine and 1st molar).

For digitization purposes, the master model was coated with Organical 3D anti-glare spray from Organical CAD/CAM GmbH, Berlin, Germany, and then scanned at the highest detail level for the stump and arc scan (3-Shape Scanner D2000, 3Shape, Copenhagen, Denmark). The resulting stereolithographic data record was the basis for all of this study’s CAM (Computer Aided Manufacturing) models. As reference standards for comparison, eight identical models were milled from a polyurethane blank (Organic Model blank, Organical CAD/CAM GmbH) in a 5-axis dental milling machine (Organical Multi Changer 20, Organical CAD/CAM GmbH). In each case, the master model was cast eight times with alginate (Tetrachrom, Kaniedenta GmbH & Co. AG, Herford, Germany) and polyether (Impregum Penta Soft/Permadyne light body, 3M Deutschland GmbH, Seefeld, Germany), and transferred into super-hard stone according to the manufacturer’s specifications (original Rocky Mountain (IV), Dental GmbH, Augsburg, Germany). 

**Figure 2:**
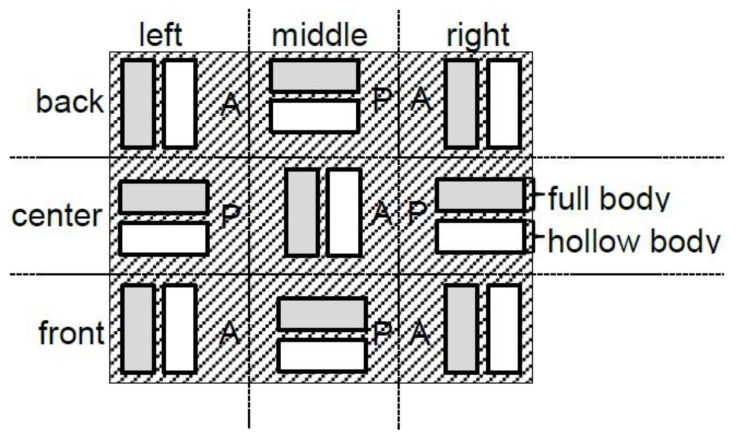
Model positioning (A: across the front; P: parallel to the front).

For the generatively produced models, the Form 2 SLA Desktop 3D Printer (FormLabs Inc., Somerville, MA, USA) was used. The stereolithographic data record was used as the basis for the solid models (F), and the Meshmixer 3D modeling program (Autodesk Research, Toronto, ON, Canada) was used to create hollow models (H), which were then sliced together with the solid models using PreForm Setup software version 2.13.1 (FormLabs Inc.). The solid and hollow models were spatially arranged in the printer as shown in Figure 2. 

**Figure 3:**
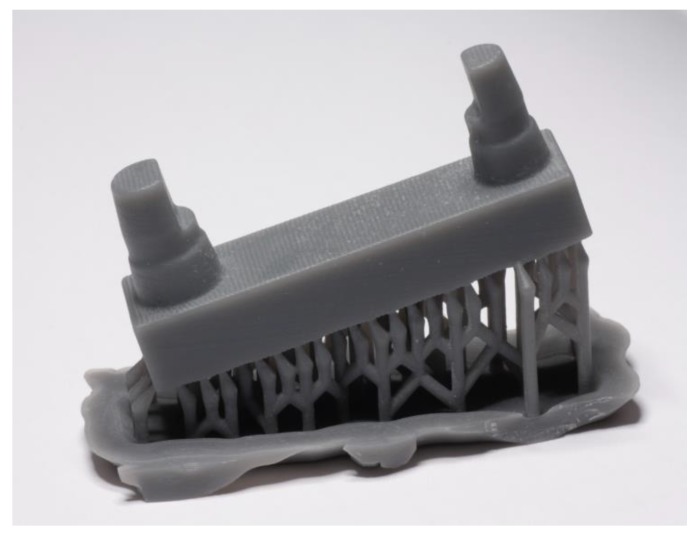
Printed model, with a layer thickness of 100 µm, angulated with a support structure.

The arrangements were always printed twice with three different layer thicknesses (S1–S3). All prints were first made with the models in a planar alignment (0°) and then with an inclination of 15° induced using support structures (Figure 3). 

Altogether, 12 printing processes were executed (D1–D12). Each printing process generated ten models aligned across the printer front (A) and eight models aligned parallel to the printer front (P). The synthetic Grey Resin V3 (FormLabs Inc.) was used for all printing processes. Table 1 summarizes the printing parameters, and Table 2 presents the resin composition. Table 3 lists the test-series sequences together with the layers calculated by the printer software, as well as the corresponding printing process times.

After the printing processes, the samples were post-processed in accordance with the manufacturer’s specifications. After removing the models from the printer, the models were rinsed with isopropyl alcohol (99.9%) to remove resin residues. For D7–D12, the support structures were removed using a miller (H251E.104.040, Gebr. Brasseler GmbH & Co. KG, Lemgo, Germany). Subsequently, two separate cleaning processes were performed using isopropyl alcohol on a vibrating unit (KaVo EWL 5442, KaVo Elektrotechnisches Werk GmbH, Leutkirch, Germany) for 10 min each. Between the cleaning processes, the released constituents were removed using compressed air (3 bar). The models were then dried at 60 °C in a drying oven (KaVo EWL TYP 5615, KaVo Elektrotechnisches Werk GmbH, Leutkirch, Germany) for 1 h. Finally, the models were post-polymerized at wavelengths of 350–500 nm for 60 min (LUXOMAT D, UVA and blue light tubes 350–500 nm in wavelength, al dente Dentalprodukte GmbH, Horgenzell, Germany).

**Figure 4:**
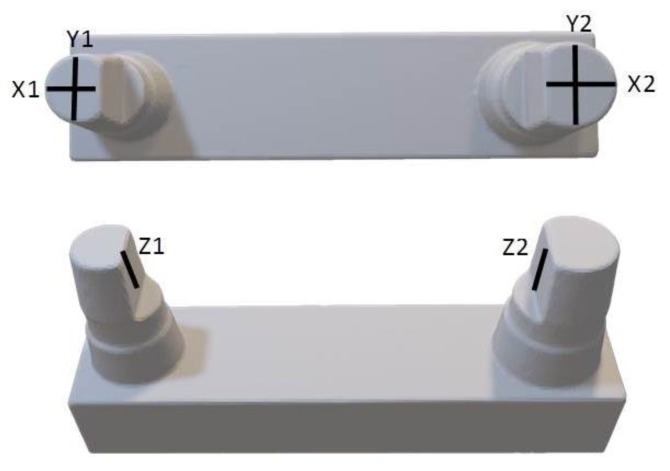
Course of the traversing lengths and measurement directions.

Until the time of measurement, the models were stored in a climate chamber at a constant temperature of 21 °C. The surface quality of each model was determined with a perthometer (MarSurf M400, MarSurf SD 26, Mahr GmbH, Göttingen, Germany), with a standard traversing length of 1.750 mm (0.250 mm × 5 mm per measurement direction). Before the measurements, the perthometer was calibrated using a roughness standard (PRN–10 Perthen-Rau-Normal, Mahr Inc., Göttingen, Germany). The surface profiles were measured over the area of the stumps in the X, Y, and Z directions (Figure 4). Each measurement was performed centrally and repeated thrice. For evaluation purposes, the mean roughness index Ra, which is automatically indicated by the device, was used [19]. The reliability of the individual measurements was determined on a sample basis using Dahlberg’s method [20].

All measured values were transferred into Excel and statistically analyzed with IBM SPSS 25 (IBM Incorp., Armonk, NY, USA). Apart from descriptive statistics, the Kolmogorov–Smirnov goodness-of-fit test was used to check the standard distribution, and Levene’s test was used to assess the homogeneity of variances. Statistical differences were verified by means of univariate Analysis of Variance (ANOVA), post-hoc tests according to Bonferroni and Tamhane, and Student’s t-test. The significance level was taken as 0.05.

## 3. Results

### 3.1. Reliability

After the repeated measurements of a test series, the measurement accuracy of the method was verified, and the error was 0.95, according to Dahlberg’s method. The Kolmogorov–Smirnov goodness-of-fit test yielded a normal distribution of all results (W ≥ 0.05). 

**Figure 5:**
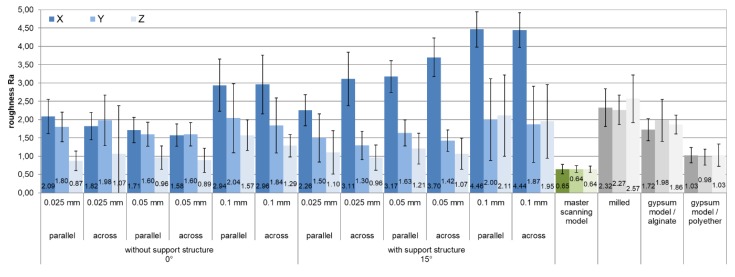
Mean values of surface roughness Ra as a function of various printing parameters in comparison with the reference models and the master model; all dimensions are in µm. X, Y, Z: measurement directions (see Figure 4, values of the measurement directions X1/X2, Y1/Y2, and Z1/Z2 were summarized); parallel/across: models’ alignment to the printer’s front (see Figure 2).

For each of the printing parameters investigated for their impacts on surface roughness, the averaged Ra values were summarized per measurement direction, as shown in Figure 5. 

The average surface roughness values of the printed models ranged from 0.87 to 4.44 µm. The milled reference models showed values between 2.32 and 2.57 µm; the cast plaster models with the alginate casting showed values between 1.72 and 1.86 µm, and those with the polyether casting showed values between 0.98 and 1.03 µm. The measurements from the master model showed continuous values of approximately 0.65 µm.

### 3.2. Ra Roughness within One Measurement Direction (X1 vs. X2, Y1 vs. Y2, Z1 vs. Z2)

The objective of the statistical analysis was to summarize the measurement values according to the measurement direction (X1 + X2; Y1 + Y2; Z1 + Z2). The model inclined at 15° resulted in different supported surfaces and even free-hanging sections inside the printer during the production process, especially in the Z area. Therefore, the values were checked for differences before summarization. Except for the X values on samples arranged across the front, the 0° samples did not show any significant differences. On the molar stumps, the surface roughness was smoother in the X direction (X2) (p < 0.01, Ra difference: 0.49 ± 0.31).

The samples inclined at 15° showed rougher surfaces at the molar stump in the Z direction (Z2) at every speed (p < 0.000, Ra differences S1: 0.43 ± 0.05, S2: 0.58 ± 0.11, S3: 1.97 ± 0.18). In contrast to the 0° samples, several of the X2 values increased in the 15° samples that were arranged parallel to the front, as well as in those arranged across the front. Accordingly, the molar stumps showed slightly rougher occlusal surfaces (p < 0.01, Ra difference: 0.44 ± 0.16). Between Y1 and Y2, no differences were verified.

### 3.3. Ra Roughness as a Function of the Measurement Direction (X vs. Y vs. Z)

Analogous to the planned procedure, the averages of the values measured along the same measurement direction were summarized. To verify significant differences among the measurement directions (X, Y, Z), the t-test was used for dependent samples. For each sample (n = 216), the differences of the paired measurement values and then the significances were calculated. 

To verify the influences of the printing parameters listed in Table 1, the number of comparisons for each parameter was set to 100%, owing to the increased amount of data. Significances (p < 0.05) were counted and set in relation to the possible number of comparisons [U].

Among measurement directions X, Y, and Z, distinct differences in the surface quality were determined. With the 0° samples (D1–D6), there were significant differences (p ≤ 0.05) in 92% of the comparisons between the X and Z directions and in 64% of those between the Y and Z directions. The roughness in the X and Y directions showed greater values (p < 0.05, X: 1.15 ± 0.47 µm; Y: 0.90 ± 0.33 µm). Between the X and Y directions, there were significant differences (p ≤ 0.05) in only 27% of the comparisons. 

In contrast, the 15° samples (D7–D12) showed significant differences (p ≤ 0.05) in 94.4% of the comparisons between the X and Y directions and in 99.07% of those between the X and Z directions. This demonstrates the rougher surface quality in the X direction compared to the Y and Z directions (X to Y: 1.98 ± 0.73 µm; Z: 2.17 ± 0.62 µm), especially at an increased printing speed (see Figure 5). Between the Y and Z directions, only 23% of the comparisons showed significant differences (p ≤ 0.05, Table 4).

### 3.4. Printing Speed

Samples that were subjected to the same printing parameters except for the printing speed, and under these conditions, congruent measurement areas showed significant deviations (p ≤ 0.05) in the surface structure for both planar and inclined models. Table 5 presents the differences among the comparisons with regard to the measurement direction and speed combination.

These results showed that the printing speed had the smallest influence on the measurement of the Y section. In contrast, except for the speed combination of 0.025 to 0.05, the measurements of the X and Z sections were significantly dependent on the printing speed. With increased printing speeds, and especially at 0.1 µm, the surface roughness mostly increased. 

### 3.5. Support Structure/Inclination

Samples were subjected to the same printing parameters except for the support structure, and when examining the influence of the model inclination, significant deviations in the surface structure could be identified in congruent measurement areas of corresponding printing processes and test-series sequences. 

There were significant differences in 91% of the comparisons in the X direction between the samples with and without a support structure. The samples with a support structure showed clearly increased surface roughness, especially at printing resolutions of 50 and 100 µm. In the Y and Z sections, the influence of the support structure was less relevant; for the samples printed with 100-µm resolution, there was no difference (p ≥ 0.05) in the Y section. In contrast, the differences in the Y section increased at a resolution of 25 µm, and under this condition, the surfaces of the samples with a support structure were smoother. The Z section showed the highest surface roughness at a printing resolution of 100 µm and 15° inclination (Figure 5). Scattered significant differences between the samples with and without a support structure confirmed the result.

### 3.6. Model Structure

Upon examining the influence of the model structure (hollow versus solid), no significant deviations in the surface structure of the planar or inclined models could be identified with regard to the position (Figure 2) in the congruent measurement areas (X, Y, Z). 

### 3.7. Model Base Orientation

Based on the understanding that the model structure did not influence the surface quality, all identically oriented samples (P vs. A) of a printing run were summarized by comparing them with regard to orientation. This comparison showed significant differences in congruent measurement directions, except for the X values in samples with a support structure, based on the increased roughness of samples oriented parallel to the front. In contrast, the X values of the samples with a support structure were mostly rougher with a vertical sample arrangement. 

### 3.8. Position Build Platform and Reprint

**Figure 6:**
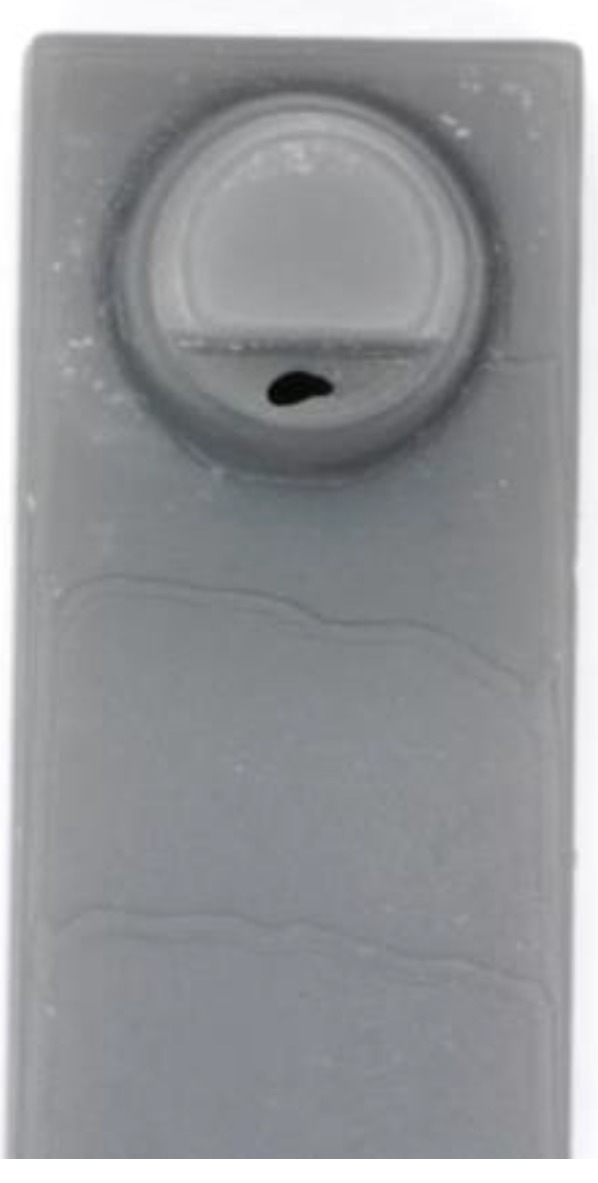
Printing defect observed in all 0° samples at the same position on the build platform.

**Figure 7:**
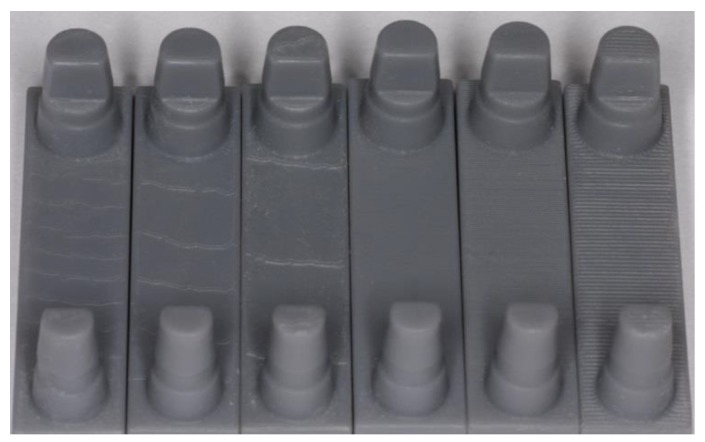
Printed models (from left: 0° and 25, 50, 100 µm; 15° and 25, 50, 100 µm). The distinct staircase-effects were found in models of lower resolution (100 µm, 3rd and 6th model from left). Models printed at 15° inclination at highest resolution showed best surfaces (25 µm, 4th from left).

Upon examining the influence of the model positioning inside the printer, no significant differences were found among identically constructed and identically arranged samples within a printing process in congruent measurement areas for either the planar or inclined models. Printing processes with identical printing parameters were repeatable (p > 0.05) only in the 0° test series (D1 and D4), and occasional (11%) significant differences (p < 0.05) were shown at the highest printing resolution level (25 µm). A defect in the shape of a hole was observed at one position on the build platform (Figure 6). All printing processes showed macroscopic characteristic surfaces, especially depending on the selected resolution and inclination (0° and 15°, Figure 7).

## 4. Discussion

Surface roughness is influenced by the manufacturing method [21]. With each manufacturing method, roughness values within a certain range are achieved only under defined circumstances. The additive manufacturing of models has the objective of creating a product with a consistently high surface quality. Sufficient quality ensures a satisfactory function [22], which in the domain of dental models, represents the fit of the denture, particularly with saw-cut models [23]. The electric profile method with mechanical contact has been shown to be effective for the quantitative evaluation of surface qualities [9,24,25,26]. 

The smaller the Ra values and deviations, the smoother the surface is. According to the recommendations available in the literature, precision level in surface qualities’ reproduction should be between 0.5 to 1 µm [7].

In this study, the master model’s Ra values of ~0.65 µm were not reproduced by any of the manufacturing methods used. However, Ra values of the plaster models transferred from alginate matched those in the literature [9,26]. In particular, the study by Johnson et al., with Ra values between 1.7 and 2.1 µm, confirms the values achieved here [9]. As expected, the plaster models from polyether castings showed the smoothest surfaces. 

According to this study’s statistical analyses, the null hypothesis had to be partly rejected. Only the printing parameters of target structure type (solid or hollow body) and the position on the build platform showed no differences with regard to the surface roughness, confirming the null hypothesis only for these two parameters. Even for these parameters, exceptions arose at spatially recurring, printer-specific defects, as shown in Figure 6. The cause may have been a contamination that disturbed the laser beam, leading to the missing polymerization of the resin at this spot. If an allocation of this position cannot be avoided, the manufacturer could eliminate defects such as holes in a post-processing step using appropriate repair sets in areas that are irrelevant to the fit [27]. 

These results show that hollow-body models could be used to save material because no differences in comparison with full-body models were verified. With regard to the application, a sufficient draining capacity for the liquid resin should be ensured to comply with the safety requirements and to avoid subsequent skin contact with resin that may remain liquid and thereby harmful. Drain holes should avoid tensions around the enclosed area caused by the so-called container effect [15,27].

**Figure 8:**
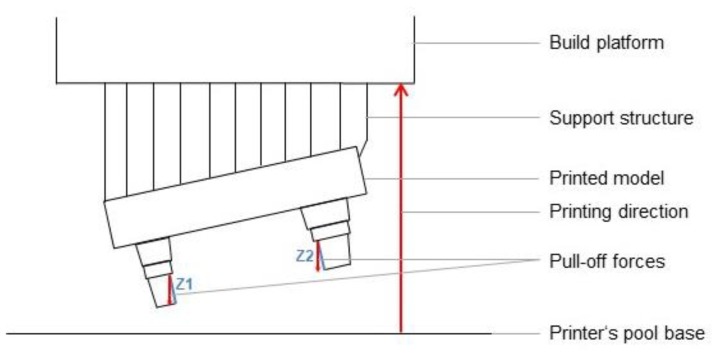
Graphical representation of the relation between pull-off forces and surface roughness in overhang areas. Z1, Z2: measurement directions in the vertical dimension.

In contrast to the master model, milled models, and plaster models (where p ≥ 0.05), the surface quality of printed models was dependent (p ≤ 0.05) on the measurement direction and the respective surface of the sample (see Figure 7). With a few exceptions (see Figure 5), the printed models showed the smoothest surfaces in the Z direction (height). At 25 and 50 µm, Ra values comparable to those of the reference models were measured. With regard to the orientation of the models, the smoothest surfaces were produced in the vertical direction, as confirmed in the literature [15]. The individual evaluations of each measured measurement direction (Figure 4), especially the comparison between Z1 and Z2 (15°), further demonstrated the influence of freely suspended areas in the build space facing the pool base. The measured values of Z2, the surface in the overhang areas, were significantly rougher than those of Z1 (p < 0.001, see Figure 8). Compared to the surfaces pointing away from the pool base, the additional pull-off forces applied to this surface when releasing the cured layer from the pool base may be the cause of this increased roughness, apart from the missing support. This assumption is supported by the increase of the effect at higher printing speeds, thicker layers, and higher resulting net weight. Depending on the laser, the polymerization processes also vary among layers with different thicknesses and, therefore, can be another cause of the significantly increased roughness at Z2 (15°) with a layer thickness of 0.1 µm [28,29,30]. In conclusion, the results indicate that overhang areas should be minimized [18].

The samples with a support structure (15° inclination) showed their highest Ra values in the X direction, particularly at the printing resolution of 100 µm. The cause for this significant increase was the macroscopically visible staircase effect (Figure 7), which occurred along gently inclined surfaces of the printed areas [14,15]. The higher the inclination, the smaller the staircase formation. According to the manufacturer, orienting large, flat surfaces to an incline of 10–20° leads to a drastically increase in success rate as the surface area of each layer as well as the amount of print contacts with the tank decrease [31]. This means that the print is subject to less force as the build platform raises with every layer [31]. Therefore other investigations recommends the even higher inclination of models by 45°, as well as the minimization of the base area [15]. In our study, a stronger inclination would have led to overhang areas and would have required additional support structures within the area of the stumps, which could have negatively impacted the measurement accuracy. Cheng et al. [15] also demonstrated that when inclination is combined with reduced layer thickness (15° inclination; 25 µm), the surfaces become significantly smoother again, and the staircase effect is considerably reduced. In this case, the surface roughness was strongly limited by the process and, thus, by the smallest possible layer thickness [32]. With regard to clinical applicability in dentistry, these models appear to be the most suitable, although the printing time for these models is considerably higher (Table 3). 

The cause of staircase formation, poor surface quality, and inaccuracies, especially on bent or inclined surfaces [13,14], must be attributed to the slicing process [33]. CAD models, often considered as reference standards in rapid prototyping technology, are mostly available in the form of stereolithographic data records [18]. From a mathematical perspective, these files represent an approximation procedure [14,18,33]. The slicing algorithm, which is determined by the printer software, cuts the component into slices of equal thickness based on the approximated triangulation data from the data record. Owing to this approach, the print is only an approximation of the original model, i.e., the end of each respective slicing layer is either within or outside the original CAD model, rather than on the exact geometry [16,33]. Errors can occur via redundant triangles, missing geometries, and misaligned facets [33]. The loss of geometry occurring in the vertical direction, called the staircase effect, is also based on the angular edges of the slicing layer, which are contrary to the modeling and on the rather parabolic shape of the layer ends during the printing process. The industry solves this problem by adjusting the slicing process accordingly, with so-called adaptive slicing, within the software programs [15,18]. The objective is to create variable rather than constant slicing layers on the surface of the component that correspond with the surface of the data record [15,28,34,35]. Therefore, instead of the fairly common assumption of edges, circular geometries are preferred for the completion of the slicing layer [15]. In order to reduce the printing time, thicker layers are printed inside a component. Other researchers have suggested working with the original CAD data or other formats [18,33]. 

With the exception of the X values at mostly higher layer thicknesses, the Ra values of the printed models corresponded to those of the plaster models, or the samples had smoother surfaces than the milled models. The much lower proportion of significant differences between measurement sections X and Y at the samples without support showed that, owing to the lack of inclination of these models, the typical staircase effect in the X direction did not develop. Thus, the staircase effect appeared to be the main cause of the difference because the differences between Y and Z were less relevant. The wave-like elevations in the samples without support (Figure 7) equally influenced the Ra values in the X and Y directions owing to their run. Consequently, especially in areas with a high accuracy, the Y values of samples with a support were lower because the measurement sections ran perpendicularly to the sample edges and, thus, on or within a stair step. With regard to practical applications, the wave-like elevations would form the basis of negative dimensional changes, and clinically, printing processes without a support could be applied only for planning models and not for precision or situational models. The causes of the elevations also appeared to depend on the software because in-house attempts to solve the problem on different printers yielded identical results. The planar sample surface was assumed to be not 100% parallel to the base of the build platform, and consequently, wave-like compensation steps, as in the staircase effect, were created in the planar case as well. Therefore, a parallel arrangement between a planar sample surface and the surface of the build platform has to be ensured for printing processes without a support, and a support structure should be selected even if uniform surfaces occur only rarely in patients’ models. To achieve better surface qualities, components should also be arranged across the front of the build platform because the roughness tended to increase on samples arranged parallel to the front. 

The significant differences that occurred only occasionally during the verification of the printing process repeatability were considered to reflect the deterioration of the transparent resin pool base, which, after repeated use, can become dull and opaque (“clouding”) [36]. This deterioration may impair the laser intensity and thus result in different surface roughness values. Therefore, the manufacturer recommends the regular exchange of the printer’s pool base [36]. As the required frequency of pool base exchange has not been defined up to now, further studies should investigate this question.

If certain recommendations are followed, 3D-printed dental models can be fabricated with comparable or higher surface quality, especially in the vertical direction, than is achieved by milling or even conventionally fabricated precision models (gypsum models made of polyether impressions, Figure 5). If dental objects do not require such high precision, as is the case with situational models, rails, drilling templates, or functional trays, the dental technician could choose positions and parameters that result in surface qualities comparable to those of conventionally fabricated gypsum situational models made of alginate impressions (Figure 5). When aiming to print with the highest possible precision, the following conditions should be ensured: arranging the models across the front of the build platform, reducing overhangs, using support structures, and selecting a high printing resolution (0.025 µm).

## 5. Conclusions

Current 3D printing applications in clinical dentistry may be inappropriate under certain printing conditions. This study’s examinations of the surface quality of models printed with the Form 2 SLA printer demonstrated the following results. First, surfaces that are significantly smoother than milled and even conventional plaster models can be produced via 3D printing under defined circumstances. Second, the model structure (solid vs. hollow) does not influence the surface quality. Third, the surface quality of printed models depends on the direction, and the inclination of the models must be established in accordance with clinical requirements. Greater precision is achieved by arranging the models across the front of the build platform, reducing overhangs, using support structures (angulating the models), and selecting a high printing resolution depending on practical requirements, although this leads to longer printing times. Fourth, the support structure and model inclination cause unavoidable staircase effects on bent or sloped surfaces, but these can be greatly reduced by adjusting other printing parameters. In general, these results show that a learning curve and appropriate knowledge are required to optimize 3D printing processes in dental applications.

Apart from the surface quality, the dimensional behavior plays an important role, especially for situational and master models, and further studies on this topic are required. Whether the above findings can be transferred to different materials and printers with different functionalities should also be investigated. The already wide application range of 3D printing in medicine and dentistry and the continuing rapid development indicates that these technologies will continue to gain in importance in these fields [1,2,3].

## Figures and Tables

**Table 1:** Printing parameters and selected (user-defined) settings.

Printing Parameters	Selected Settings
Printing speed (layer thickness)	0.025 slow (layer thickness: 25 µm) [S1]
	0.05 standard (layer thickness: 50 µm) [S2]
	0.1 fast (layer thickness: 100 µm) [S3]
Inclination (support structure)	0° (without support structure)
	15° (with support structure)
	(canine up, molar down)
Model structure	hollow body [H]
	solid (full) body [F]
Model base orientation (see Figure 2)	model base: parallel to the front [P]
	model base: across the front [A]
Position build platform (see Figure 2)	front area (right, middle, left)
	center area (right, middle, left)
	back area (right, middle, left)

**Table 2:** Composition of Grey Resin V3.

Ingredient	%
Methacrylated oligomer	≥75 ~ ≤90
Methacrylated monomer	≥25 ~ ≤50
Diphenyl (2,4,6-trimethylbenzoyl) phosphine oxide	≥1 ~ <3

**Table 3:** Printer-calculated material-specific parameters and the sequence of tests *.

Test-Series Sequences	Printing resolution/Support Structure Inclination	Number of Layers	Print Time
D1 and D4	25 µm/0°	659	466 min
D3 and D6	50 µm/0°	330	249 min
D2 and D5	100 µm/0°	165	146 min
D8 and D11	25 µm/15°	1036	620 min
D9 and D12	50 µm/15°	564	381 min
D7 and D10	100 µm/15°	314	285 min

* The consecutive numbering of the printing processes indicates the printing sequence, selected with regard to the layer thickness and use of a support structure.

**Table 4:** Significances of roughness value Ra comparisons among measurement directions.

Model Structure	Position Build Platform	Comparison Measuring Direction	D1	D2	D3	D4	D5	D6	D7	D8	D9	D10	D11	D12
Full body	Back left	X/Y	0.120	0.232	0.297	0.531	0.001	0.161	0.195	0.000	0.000	0.000	0.000	0.000
		X/Z	0.000	0.006	0.000	0.004	0.000	0.121	0.005	0.000	0.000	0.002	0.000	0.000
		Y/Z	0.000	0.084	0.000	0.003	0.016	0.050	0.250	0.509	0.253	0.096	0.020	0.557
	Back right	X/Y	0.912	0.001	0.285	0.493	0.016	0.128	0.002	0.008	0.000	0.024	0.000	0.000
		X/Z	0.003	0.008	0.001	0.000	0.007	0.001	0.006	0.000	0.000	0.004	0.015	0.000
		Y/Z	0.011	0.510	0.001	0.000	0.178	0.001	0.778	0.069	0.331	0.703	0.092	0.137
	Center middle	X/Y	0.394	0.009	0.130	0.682	0.023	0.610	0.021	0.001	0.000	0.000	0.000	0.000
		X/Z	0.001	0.001	0.001	0.000	0.000	0.029	0.00	0.000	0.000	0.000	0.003	0.000
		Y/Z	0.000	0.208	0.000	0.000	0.007	0.033	0.676	0.025	0.328	0.100	0.381	0.041
	Front left	X/Y	0.625	0.019	0.686	0.198	0.256	0.597	0.000	0.000	0.002	0.000	0.000	0.000
		X/Z	0.001	0.007	0.012	0.028	0.005	0.005	0.000	0.000	0.003	0.000	0.000	0.000
		Y/Z	0.000	0.051	0.000	0.006	0.251	0.003	0.795	0.026	0.206	0.502	0.008	0.213
	Front right	X/Y	0.503	0.011	0.119	0.896	0.046	0.357	0.056	0.000	0.000	0.000	0.001	0.000
		X/Z	0.000	0.003	0.000	0.130	0.008	0.021	0.000	0.000	0.000	0.000	0.001	0.000
		Y/Z	0.000	0.205	0.006	0.129	0.041	0.013	0.082	0.867	0.052	0.711	0.002	0.009
	Back middle	X/Y	0.365	0.004	0.196	0.292	0.115	0.254	0.024	0.000	0.000	0.000	0.001	0.002
		X/Z	0.000	0.015	0.001	0.055	0.034	0.000	0.001	0.000	0.000	0.004	0.000	0.000
		Y/Z	0.001	0.699	0.000	0.036	0.134	0.000	0.633	0.212	0.007	0.474	0.003	0.074
	Left middle	X/Y	0.701	0.006	0.765	0.104	0.070	0.231	0.008	0.557	0.000	0.000	0.004	0.001
		X/Z	0.000	0.014	0.016	0.002	0.003	0.002	0.028	0.618	0.002	0.014	0.015	0.000
		Y/Z	0.001	0.084	0.006	0.001	0.300	0.033	0.673	0.882	0.382	0.132	0.135	0.017
	Right middle	X/Y	0.167	0.173	0.831	0.374	0.001	0.451	0.001	0.132	0.000	0.000	0.001	0.000
		X/Z	0.008	0.006	0.005	0.000	0.068	0.285	0.000	0.000	0.000	0.000	0.000	0.003
		Y/Z	0.007	0.463	0.001	0.001	0.846	0.429	0.628	0.134	0.125	0.350	0.008	0.437
	Front middle	X/Y	0.188	0.014	0.039	0.636	0.415	0.711	0.036	0.002	0.000	0.121	0.000	0.001
		X/Z	0.005	0.004	0.001	0.000	0.000	0.025	0.003	0.010	0.000	0.001	0.000	0.000
		Y/Z	0.002	0.158	0.000	0.000	0.221	0.022	0.593	0.124	0.071	0.320	0.004	0.017
Hollow body	Back left	X/Y	0.001	0.000	0.244	0.002	0.325	0.910	0.000	0.003	0.000	0.000	0.000	0.000
		X/Z	0.002	0.002	0.002	0.004	0.004	0.001	0.002	0.000	0.000	0.005	0.000	0.000
		Y/Z	0.000	0.867	0.000	0.002	0.047	0.003	0.176	0.001	0.063	0.189	0.590	0.374
	Back right	X/Y	0.384	0.008	0.415	0.589	0.064	0.654	0.000	0.001	0.000	0.001	0.000	0.001
		X/Z	0.005	0.006	0.046	0.004	0.017	0.000	0.005	0.000	0.001	0.011	0.001	0.001
		Y/Z	0.004	0.258	0.150	0.004	0.251	0.000	0.303	0.134	0.416	0.225	0.280	0.248
	Center middle	X/Y	0.061	0.009	0.417	0.077	0.076	0.149	0.005	0.000	0.000	0.010	0.001	0.000
		X/Z	0.001	0.001	0.005	0.175	0.006	0.087	0.004	0.000	0.000	0.006	0.000	0.000
		Y/Z	0.017	0.301	0.018	0.282	0.125	0.339	0.942	0.121	0.119	0.638	0.192	0.065
	Front left	X/Y	0.202	0.005	0.019	0.151	0.030	0.913	0.001	0.000	0.000	0.000	0.000	0.000
		X/Z	0.490	0.002	0.019	0.011	0.003	0.006	0.000	0.000	0.000	0.000	0.001	0.000
		Y/Z	0.549	0.123	0.014	0.006	0.025	0.010	0.453	0.685	0.116	0.000	0.008	0.600
	Front right	X/Y	0.491	0.011	0.412	0.783	0.000	0.727	0.000	0.000	0.000	0.000	0.002	0.000
		X/Z	0.001	0.001	0.102	0.001	0.003	0.062	0.000	0.000	0.000	0.000	0.000	0.000
		Y/Z	0.002	0.047	0.176	0.011	0.121	0.043	0.522	0.329	0.034	0.090	0.003	0.031
	Back middle	X/Y	0.034	0.093	0.691	0.007	0.016	0.276	0.000	0.011	0.000	0.024	0.000	0.016
		X/Z	0.000	0.003	0.001	0.005	0.009	0.002	0.001	0.000	0.000	0.001	0.000	0.000
		Y/Z	0.006	0.299	0.013	0.043	0.597	0.002	0.393	0.023	0.018	0.565	0.078	0.122
	Left middle	X/Y	0.272	0.009	0.399	0.018	0.353	0.812	0.000	0.867	0.000	0.000	0.001	0.000
		X/Z	0.004	0.012	0.025	0.000	0.035	0.001	0.008	0.022	0.000	0.016	0.006	0.000
		Y/Z	0.000	0.748	0.032	0.001	0.277	0.000	0.245	0.111	0.195	0.372	0.052	0.196
	Right middle	X/Y	0.526	0.005	0.732	0.000	0.564	0.823	0.031	0.004	0.000	0.000	0.001	0.000
		X/Z	0.001	0.026	0.000	0.000	0.001	0.035	0.001	0.003	0.000	0.000	0.000	0.000
		Y/Z	0.024	0.202	0.007	0.000	0.053	0.119	0.520	0.408	0,006	0.108	0.007	0.842
	Front middle	X/Y	0.436	0.159	0.523	0.076	0.189	0.254	0.012	0.000	0.000	0.003	0.003	0.000
		X/Z	0.001	0.031	0.035	0.001	0.001	0.028	0.010	0.000	0.000	0.000	0.000	0.000
		Y/Z	0.004	0.100	0.061	0.000	0.072	0.092	0.774	0.334	0.001	0.847	0.047	0.050

**Table 5:** Significances (p ≤ 0.05) within measurement directions depending on the selected printing speed.

Combination of Printing Resolutions	15°			0°		
	X	Y	Z	X	Y	Z
0.025 to 0.05	78%	11%	22%	39%	22%	6%
0.025 to 0.1	100%	22%	94%	94%	0%	89%
0.05 to 0.1	89%	6%	72%	100%	11%	84%
